# Cancer Phenotype Diagnosis and Drug Efficacy within Japanese Health Care

**DOI:** 10.1155/2012/921901

**Published:** 2012-05-22

**Authors:** Toshihide Nishimura, Harubumi Kato, Norihiko Ikeda, Makoto Kihara, Masaharu Nomura, Yasufumi Kato, György Marko-Varga

**Affiliations:** ^1^Department of Surgery, Tokyo Medical University, 6-7-1 Nishi Shinjuku Shinjuku-ku, Tokyo 160-0023, Japan; ^2^Graduate School of Medical Science, Yokohama City University, 3-9 Fukuura Kanazawa-ku, Yokohama, Kanagawa 236-0004, Japan; ^3^Medical ProteoScope, 1-6 Suehiro-cho, Tsurumi-ku, Yokohamashi, Kanagaewa, 230-0045, Japan; ^4^Thoracic Surgery, Niizashiki Chuo General Hospital, 7-2 1-chome, Tohoku, Niiza, Saitama 352-0001, Japan

## Abstract

An overview on targeted personalized medicine is given describing the developments in Japan of lung cancer patients. These new targeted therapies with novel personalized medicine drugs require new implementations, in order to follow and monitor drug efficacy and outcome. Examples from IRESSA (Gefitinib) and TARCEVA (Erlotinib) treatments used in medication of lung cancer patients are presented. Lung cancer is one of the most common causes of cancer mortality in the world. The importance of both the quantification of disease progression, where diagnostic-related biomarkers are being implemented, in addition to the actual measurement of disease-specific mechanisms relating to pathway signalling activation of disease-progressive protein targets is summarised. An outline is also presented, describing changes and adaptations in Japan, meeting the rising costs and challenges. Today, urgent implementation of programs to address these needs has led to a rebuilding of the entire approach of medical evaluation and clinical care.

## 1. Health Care Costs and Impact

The rising cost in Japanese healthcare system, with an elderly population that is expected to reach 30% of the population by 2020, is a major challenge to the health care system (http://www.ipss.go.jp/syoushika/tohkei/Popular/Popular2011.asp?chap=0 in Japanese). This growth of elderly population in society is a trend that can be seen in other countries as well. The US, for instance, has an estimated growth of 16%, and Germany 23% by 2020. However, the Japanese situation is extreme in that 39.6% is predicted by 2050, which is far more than any other country in the world [1]. At the moment, Japan has most probably the largest future costs associated to the increasing elderly population. At the same time, the country has one of the lowest medical health care spends (in comparison to other developed countries) which is about 8.1%. This part of the budget is used on the national medical expense, based on the national GDP. These anticipated future costs and changes in society would be challenging the Japanese society for decades to come. To meet these alterations, it is envisioned that major changes will be implemented in emerging technologies and patient treatment procedures [2].

It is clear from a historical background that the future of biomedical sciences will be driven by the ability to adopt novel technologies, which will generate huge amounts of data outputs from clinical samples. One major consequence will be to utilize the new technology deliveries as the basis to understand the disease complexity and to develop new treatments. This is especially relevant to diseases such as lung cancer (LCa) and chronic obstructive pulmonary disease (COPD), the latter, a disease that is rapidly increasing and that presents itself in combination with LCa. These pulmonary diseases currently carry a huge mortality and cost to the health care system. At the same time, these diseases have been shown to advance prognosis and reduced cost to healthcare system by early detection, prescription of personalized medicine, and evaluation of response to treatment. These diseases are known to be highly complex and multifactorial. It is not possible at this stage to assign a single molecule related to one disease or clinical complaint. On the contrary, there are hundreds (multiple signals), and there is a need of selecting from multiple signals. This is a highly demanding task, as this is hampered by the lack of tools and data for early diagnosis. In addition, modeling of disease progression and evaluation of treatment response is also something that the science community is still working on, and not a scientific tool that is available. LCa and COPD are both known to cluster in families and are more common in elderly population. Aggregation has been observed in families which would suggest a genetic or an environmental connection. Pathologically it has been observed that a lower lung function is seen in COPD patients, which would indicate a significant risk and a valuable predictor of incidents in lung cancer. We are experiencing that prevalence is increasing in patients with lung cancer, which is independent of age, sex, and smoking history. Consequently, there is a sixfold higher prevalence in lung cancer patients.

Currently, these disease areas are facing major challenges where major research resources are directed, such as the stratification of phenotypes along with an early indication of disease and diagnostics that can identify disease appearance and staging. With an optimal treatment, based on individual medical needs, is currently fundamental to the rebuilding of the entire medical and clinical system. This will be including the cases of lung tumors, their diagnosis, the surgical treatments, and/or chemotherapy of each individual subject.

In this respect, the concept of personalized medicine declared as a working proposition still is in its initial phase of developments and implementation worldwide. A major and unremitting effort is considered necessary to achieve these developments such as the requirement to establish the ranges of quantitative assays. These assays need to be able to separate healthy from diseased individuals in a variety of basic sciences such as genomics, proteomics, and metabolomics, as well as clinical sciences. Clinically, surveillance, diagnosis, and treatment should be in focus, which then is applied to match scenarios of individual disease with best practice individual treatment efficacy. Lately, a major focus of the introduction of targeted personalized medicine is marker associations to drug efficacy and safety. The targeted treatments are reducing costs for an aging Japanese population.

## 2. Cost and Benefits for the Japanese Patients

In many respects, Japan has been pioneering the optimal use of drugs for the Japanese population. This is especially highlighted by the successful use of personalized medicines for lung cancer treatments. The epidermal growth factor receptor (EGFR) tyrosine kinase inhibitors (TKIs) are by now well-established treatment for advanced non-small-cell lung cancer (NSCLC). The advantage with these new generation of targeted drugs is that they in comparison to chemotherapy show that they are typically well tolerated and without cytotoxic side effects. The discovery of a novel class of epidermal growth factor receptor-tyrosine kinase inhibitors (EGFR-TKIs) was first made by AstraZeneca in 1994. EGFR is a target that is overexpressed in high levels on cancer cell surfaces in general, and particularly on non-small cell lung cancer (NSCLC) cells. Consequently, elevated levels of EGFR have been linked with progressed disease, cancer spread, and poor clinical prognosis. The tyrosine kinase enzyme(s) in the EGF receptor is inhibited by Gefitinib [3]. It results in a blocking effect of signaling that is linked to the functions of growth and spread of tumors. These types of tumors are predominantly effective with IRESSA treatments. The specific action of Gefitinib is linked to a high-affinity binding to the mutated EGFR tyrosine kinase domain with high specificity. A significant tumor shrinkage upon IRESSA treatment occurs in the majority of patients with EGFR mutation.

**Figure 1:**
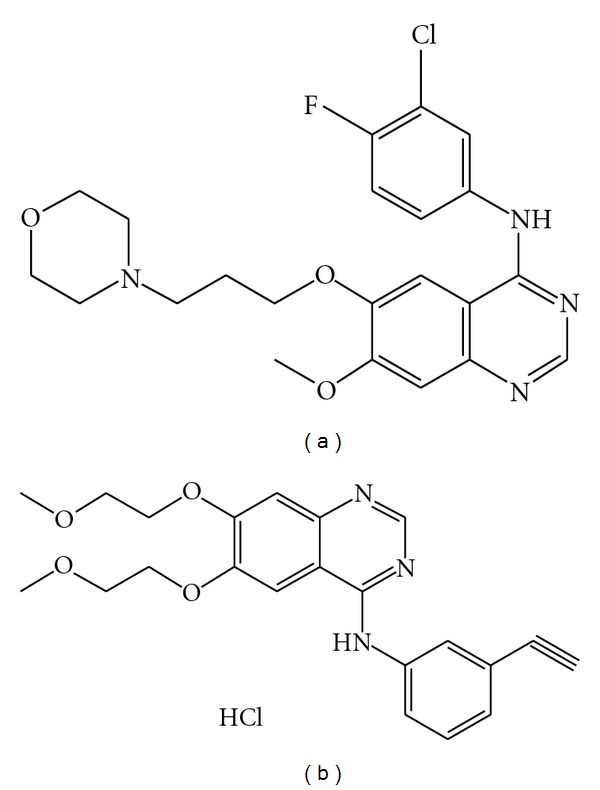
Chemical drug structure of Gefitinib, as a freebase (a) and Erlotinib as a (HCl) salt (b), respectively.

In this respect, both IRESSA (Gefitinib) and TARCEVA (Erlotinib) have been used by Japanese patients between 2002 and 2007, respectively, for treatment of advanced NSCLC [4]. AstraZeneca was the first pharma company that managed to get the targeted TKI, small molecule drugs to efficiently treat lung cancer patients. The chemical properties of Erlotinib and Gefitinib drug compounds are somewhat similar, while the chemical structures vary significantly, as shown in Figure 1.

These targeted small molecule drugs are used as oral monotherapy treatments. They have been proven to offer a superior quality of life compared with doublet chemotherapy (carboplatin/paclitaxel) as first-line treatment for EGFR mutation-positive advanced NSCLC. The personalized therapy approach was also recently proven to be applicable, not only to Japan, but also to Asians in general.

In Japan, rising costs have impacted on the framework of maintaining an efficient and effective healthcare system. Today, urgent implementation of programs to address this need has led to a rebuilding of the entire approach of medical evaluation and clinical care. Central to this realignment is the concept and practice of providing personalized medicine as an effective means for delivering effective care.

Currently, there are some reports on the costs involved in patient treatments utilizing patient diagnosis upon drug use that ultimately relate to the issue of responders versus nonresponders. This is an important consideration that gets more and more attention since the overall economy, that society needs to provide, is related to the costs of personalized medicines, which in most cases are significantly higher than many other traditional drugs. Davis et al. recently presented new developments and experiences that increasingly confirm the value of using personalized medicines [5]. From this paper, it has been concluded that protein- and genetic-biomarker diagnosis show increasingly high cost-effectivity, in providing the right medicine to the right patient at the first prescription. Recent medical savings was reported, where $40–80,000/year and patient is saved by the introduction of personalized medicine [5]. Safety considerations regarding possible side effects are also an added value, when patients show a negative drug test [6, 7].

As Japan currently is the world's largest market for tobacco products, consequently smoking related lung cancer's mortality has already been the highest among all cancers. These life conditions will have a severe impact on the quality of life of the smoke-induced and -related diseases in Japan. These effects are probably higher in Japan than in any other country, although the situation in China is alarming as well. In China, the combination of smoking, organic cooking, and the environmental factors are key drivers of lung cancer and COPD.

The tough challenge that the physician is faced with is to treat with an effective drug, and examples of these challenges have been reported and presented on at international congresses by our group over the years. The lung cancer phenotype is also of mandatory importance to correctly diagnose the cancer variant. Standard computer tomography (CT) imaging could efficiently be complemented by protein biomarker assays, such as multiplexing multiple reaction monitoring (MRM) assays [8, 9]. In addition, a multicenter study recently presented the solidity of the MRM technology platform [10].

Looking into the pipeline of coming drug products, currently in clinical phase trials, the expectation of new, targeted medicines, as well as antibody-based biopharmaceuticals, is expected to grow considerably. Biopharmaceutical medication such as in the treatment of NSCLC patients [11] revealed that Bevacizumab, a monoclonal antibody against vascular endothelial growth factor, has been shown to benefit patients with non-small-cell lung cancer resulting in a significant survival benefit [12].

The total market of targeted anticancer drugs in Japan, Europe, and United States has been continuously increasing which exceeded more than US$28 billion. Especially, regarding antibody drugs, their size in 2007 was around US$36 billion and estimated to increase up to US$67 billion in 2013. In Japan, about 10 antibody drugs were currently in market, which cover a total of 1 billion US$ (http://www.seedplanning.co.jp/press/2008/0610.html).

Personalized drugs have a natural Ying & Yang partnering effect within the treatment concept, which is the diagnostic marker that can assign an optimal drug treatment strategy that will benefit both the patient as well as the taxpayers.

The future optimal concept that healthcare institutions as well as politicians and industry are looking for is the following:

*“The Right Medicine to The Right Patient at the Right Time Point.”*

## 3. Impact to Society of Personalized Medicines

Genetic alterations of EGFR (exons 18, 19, 20, and 21) are important for predicting the efficacy of personalized medicine such as IRESSA in patients with lung cancer [13, 14].

The observation of EGFR somatic mutations in Japanese patients was made at a time point during the phase III study with IRESSA in North America [15]. The phase III study was not able to show a statistical significance of the drug with the criteria that were set at the time. However, previously, in the phase II study, the study outcome was highly successful with the dose and conditions given at the time, why AstraZeneca was urged to start production and distribution to patients before the phase III study was finalized. This was also the action that AstraZeneca took at the time. Nonetheless, at this stage, the subset of the Asian part of the patient cohort did prove a significant outcome upon IRESSA treatment, why the mode-of-drug action already at this time point was suspected to be related to EGFR mutation. The test to analyze the EGFR mutation status was developed that could identify one phenotype: mutated versus nonmutated [13]. These findings were associated with a long path of mechanistic development works. The resulting data from these studies and additional followup investigations resulted in a standard procedure using the EGFR-mutation assay, which could detect mutations in exon18 (G719A/C/S), exon19 (E746-A750 deletion, L747-P753 deletion insertion S), exon20 (S768I), and exon21 (L858I, L861Q) more than 1% mutation rate in the clinic today. In Japan, a positive indication will result in personalized medicine prescriptions [13, 16].

**Figure 2:**
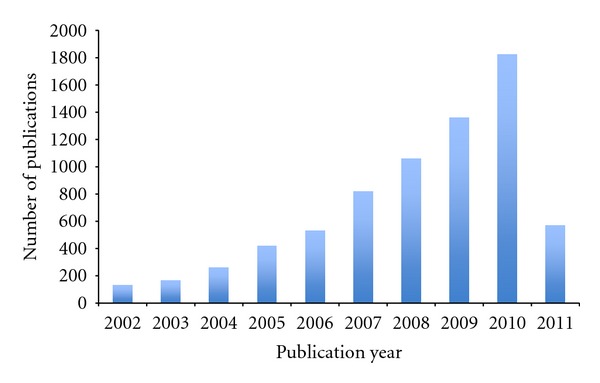
Publication frequency during the past 10 years using the keyword “clinical biomarker” in search of ISI Web of Knowledge on April 29, 2011.

In protein expression research today, we discover, develop, and validate proteomics findings for new prospect applications in a clinical setting. As there is increasing funding globally available for research programs that can improve the diagnosis and stratification of patients, as well as biomarkers for both safety assessments and efficacy, an increasing number of studies and study data that illustrate these developments are being published, as outlined in Figure 2.

Gefitinib (IRESSA, ZD1839) was developed as a specific inhibitor of the EGFR tyrosine kinase. Gefitinib targets the EGFR for therapeutic drug intervention within lung cancer. Early reports appeared on the experience of lung cancer patients with EGFR mutations. These were among the first clinical data that provided correlation with clinical response to Gefitinib therapy [16]. Personalized medicines have an improved efficacy over chemotherapy and radiation treatments. While targeted drugs can reach 70–75% efficacy, the combined effects of LCa chemotherapy and radiation will reach about 35%. With respect to the well-being of patients, the targeted drugs are not only more efficient but also provide an improvement in quality of life.

Recently a clinical study in four Asian countries was conducted: the IRESSA Pan-Asia Study, the “IPASS” study, in which 1,217 NSCLC patients were enrolled [17]. The IPASS study was an open label, randomised, parallel-group study that assessed the efficacy, safety, and tolerability of IRESSA versus doublet chemotherapy (carboplatin/paclitaxel) as first-line treatment in a clinically selected population of patients from Asia.

The adenocarcinoma cohort of the study with Japanese patients was enrolled for efficacy biomarker discovery. The ultimate objective is to identify responders to Gefitinib treatment to nonresponders. The final outcome for diagnostic prediction is still under investigation.

## 4. Clinical Biomarkers

There is a lack of protein biomarker and imaging diagnostics today within lung disorders, such as LCa and COPD. These new clinical tools are expected to be used as early indicator of disease, or as personalized indicator assays for targeted and stratified disease phenotype drug treatments in the near future. There is also a poor understanding of the mode of drug action mechanisms, by commonly used therapies, which is also true for new drugs introduced to the market. The actual targeted cells and proteins within disease and the actual drug interactions are by no means understood for most medicines used in today's therapies. These drug characteristics are needed for both efficacy and safety improvements and also requested by regulatory authorities, like FDA, MLHW, and EMEA.

Using a multiple biomarker approach such as proteomics (the simultaneous study of large parts of the human proteome to give a global view of differential expression of proteins in blood or tissue), rather than simply a conventional single biomarker, potentially increases predictive power both through increased robustness deriving from multiple measurements and the opportunity to combine information from multiple biological processes. To support high-quality generation of such information, we combined in a novel way several key study components: robust study design, well-defined phenotypic definitions, careful sample collection procedures, stable advanced liquid-chromatography (LC-) tandem mass spectrometry, MS/MS-based peptide separation and detection methods, and statistical analysis incorporating proteomic and clinical information. We developed stringent methods for database protein annotation of detected peptide peaks and biological interpretation using literature mining software, plus extensive quality control and validation in lung cancer studies. The first presentation on IRESSA biomarkers was presented at the European Cancer meeting (ECCO, Paris, France) in 2007 (http://www.ncbi.nlm.nih.gov/pubmed/16381621).

It is also expected that clinical chemistry diagnosis with biomarkers will become highly valuable as a screening platform with speedy and cheap diagnosis assays in relation to imaging, where, for instance, a typical pricing for CT or magnetic resonance imaging (MRI) in Japan is in the order of $10,000.

Mass-spectrometry-based proteomic approaches are based on digital molecular recognition (*m/z, mass over charge*) and sequencing in high resolution, and so those investigations of lung cancer have thrown light on understanding complexity of patient disease status, and from the clinicians' point of view, we believe that a utilization of useful biomarkers based on their quantitative molecular expressions can stratify patients and improve their therapeutic strategies for better clinical outcomes, and that such approaches would diminish unnecessary multiple treatments, which shall contribute to reduction of medical healthcare costs.

As the first country in the world, Japan has declared a pricing strategy that includes demands for biomarker diagnostics that should be used in combination with new drugs introduced into the market [18, 19]. Thereby, a targeted treatment with patient stratification will be achieved, with phenotype selection for responders to drug treatment [20].

In this declaration, pharmacogenomic and proteomic technologies are promoted in the discovery and development of drug-related biomarkers by the drug pricing committee within the MHLW (http://www.mhlw.go.jp/shingi/2009/07/dl/s0715-9a.239.pdf).

The overall aim of this declaration is to reach a Japanese pricing strategy that will be used in order to promote safe and efficient approved drugs for the treatment of Japanese patients.

### 4.1. Drug Safety Biomarker Studies

Stratification of patients who will benefit from Gefitinib treatment or even suffer side effects is major medical concern. A large Gefitinib postmarketing surveillance study in Japan (3,322 patients) reported 5.8% in rate of intestinal-lung-disease-(ILD-) type events. To reveal risk factors of ILD occurrence, a large-scale plasma proteomic study has been conducted in a cohort of NSCLC patients treated with Gefitinib [18, 19].

Reports have been coming from Japanese patients with non-small-cell lung cancer, treated with Gefitinib, that events of ILD appear some weeks after first administration of drug. ILD is a highly heterogeneous pathophysiology state that is not easily diagnosed.

ILD will affect the lung parenchyma and/or the alveolar region [21–23]. It was also presented by Kudoh's group that a high prevalence of drug-induced pneumonia in Japan has been identified in several studies [24–27]. Specific Gefitinib-related ILD occurrence has also been reported in a number of studies, where the occurrence frequency and predictive factors have been investigated [24–27].

A number of reports have been made over the last 4 years, where Gefitinib treated patients developing ILD were compared to a control group where the patients did not develop ILD [18]. In 2009, preliminary data were presented on safety biomarkers at the European Proteomics Association Congress (Stockholm, Sweden) from Japanese lung cancer patients undergoing IRESSA treatment. (http://www.eupa.org/EuPA2009/proceedings/6.Posters/2.%20Biomarker%20Proteomics%20and%20Applications%20%2874%29/P2-43.pdf).

The biomarker candidates were presented for the first time at the 26th ISPE Congress (International Conference on Pharmacoepidemiology and Therapeutic Risk Management) in 2010 in Brighton, UK (http://www.pharmacoepi.org/meetings/26thconf/26th_ICPE_Final_Program.pdf).

**Figure 3:**
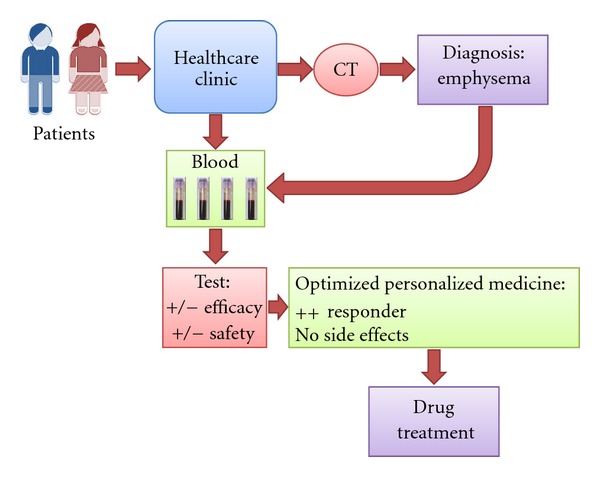
Diagnostic testing linked to targeted personalized drug treatment of lung cancer patients.

 Recent followup patient analysis with plasma samples from the respective patient groups (CASES and CONTROLS of ILD) was analyzed by shotgun sequencing, utilizing the LC-MS proteomics platform [28, 29]. Typically, tens of thousands of mass signals generated from expressed protein sequences were detected, from where the differential expression analysis was performed. In parts of these studies, there was like ~7 million sequence data generated, with MS/MS fragmentations [30]. This is probably the largest clinical protein biomarker discovery study ever performed in the industry where personalized medicine treatments were made. The resulting outcome, in terms of protein sequence output and the clinical data within the IRESSA repository database, must also be one of the largest ever undertaken [30]. The future targeted drug treatments inking efficacy with safety was pioneered in Japan, and the improved quality of life for patients in addition to the cost benefit society will gain is illustrated in Figure 3. 

### 4.2. Specific Lung Cancer Phenotype Markers

Proteomic investigations of lung cancer use clinical materials such as frozen and formalin-fixed paraffin-embedded (FFPE) tissue specimens. Laser microdissecting the cancer cells from the pulmonary patient tissues is an efficient way to make: (i) drug-specific target discovery and (ii) biomarker discovery studies. It allows for expert pathological identification of the tumor cells in the tissue compartments, which is made by an electronic image analysis report. The areas within the tissues are marked by the pathologist. Next, the operator can use the marked image in order to make the laser micro-dissection and tumor cell isolation. This strategy allows for expert utility, where the experimental operators are distantly separated geographically.

Biomarker diagnosis studies were undertaken on large cell neuroendocrine carcinoma (LCNEC) patients, applying laser-microdissected tumor cell isolates [31]. LCNEC is a rather new lung cancer phenotype and has been categorized into one of the subtypes of large-cell carcinoma (LCC) [32]. The LCNEC phenotype, thus, was demonstrated to develop high-grade neuroendocrine tumors. Classifying the LCNEC phenotype is highly challenging, and as of now, there is no definite treatment for LCNEC patients. As LCNEC has not been found to be highly amenable to chemotherapy, as compared to small-cell lung carcinoma (SCLC), a diagnostic test is needed in order to separate the LCNEC patients from LC and SCLC. An additional complication is also that currently there is no specific marker for LCNEC that accurately identifies the disease evolvement, which allows for a targeted treatment strategy.

FFPE tissues have been archived in hospitals worldwide, together with detailed clinical records, for example, disease history, clinical examination results, drug response, and adverse reaction of individual patients. Recently, emerging technology for FFPE tissue proteomics has made it possible to study protein expression using FFPE tissue specimens, providing a great opportunity for biomarker discovery using clinically archived FFPE tissues accompanied by both definitive diagnoses and known clinical outcomes [18, 19, 28, 33–35]. Our proteomic studies utilizing the laser-microdissected FFPE tissues of LCNEC, LCC and SCLC, resulted in more than 100 significant protein biomarker candidates [28]. Lately, MRM multiplex assay has become popular due to their generic applicability [36, 37]. The MS-based verification study of these candidates has been conducted by recent developments using MRM quantitative mass spectrometry as a novel methodology [29].

Following validation of biomarkers, MRM offers quantifications of proteins in complex biological matrices where key protein sequences are targeted within the assay [38]. In combination with appropriate stable isotope-labeled internal standards, the MRM assay technology provides absolute quantitation of the biomarkers. A great advantage is that a high number of proteins of interest can be monitored simultaneously within the MRM assay cycle.

MRM quantifications present high sensitivity and speed, which is a future requirement. High-throughput screening of clinical samples for candidate biomarkers within the clinical study area is the next frontier, where the MRM technology is developing further.

**Figure 4:**
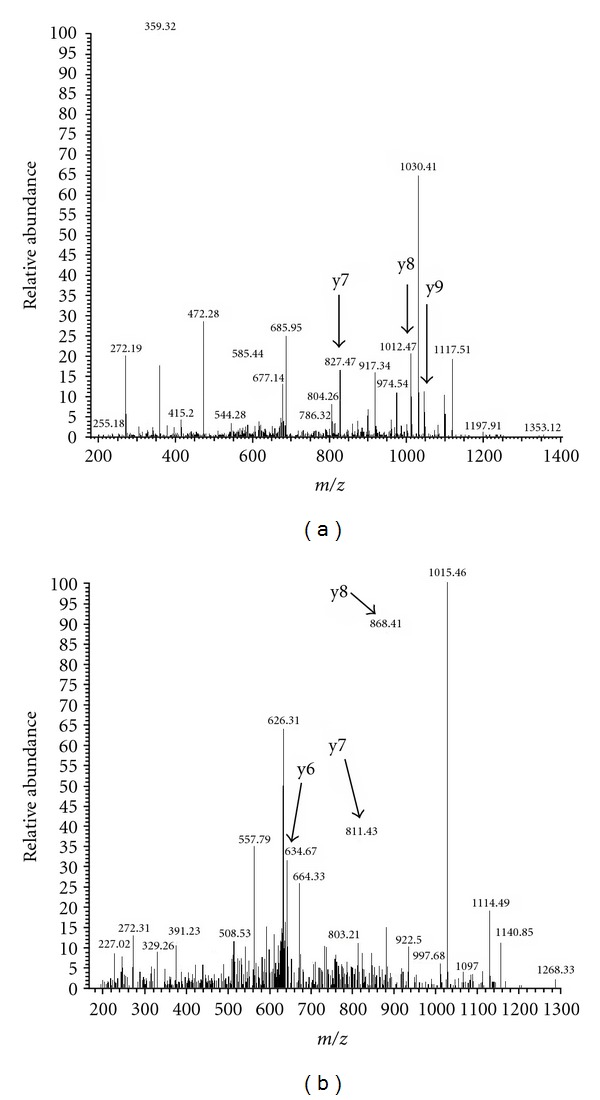
Mass spectra on the sequence identity of (a) Stathmin and (b) major vault protein (MVP) that was found differentially regulated in the LCNEC patient tissues.

Our MS-based quantitative studies of those candidates have verified 44 promising biomarker candidates, using MRM [28].  Figure 4 shows the corresponding MS/MS spectra of (a) Stathmin and (b) major vault protein (MVP) from the corresponding MRM quantitation readouts. We discovered that Stathmin is highly expressed in neuroendocrine lung tumors (within the SCLC and LCNEC patient groups), and that MVP would be significant to LCC [18, 19]. It is expected that their subset of candidates would be useful for an improved differential diagnosis of LCNEC patients. These studies are undertaken by our research team as well as others throughout almost a decade. The studies undertaken in Japan are all performed in a clinical environment with close collaboration to the expertise of hospitals. The surgeons have provided the bed-side resected tissue samples for our studies, along with clinicians, that have been giving their experienced guiding for biofluid sampling, CT-imaging, and clinical demography data. In addition, we have been collaborating closely with pathologists who have performed the diagnosis of patients and been instrumental in our own proteomic studies, when comparing classical histology with proteomics-generated biomarker diagnosis predictions.

In view of this, the recently approved Neuroendocrine Lung Cancer group, which in terms of diagnosis is a highly challenging lung cancer phenotype to verify, will have novel biomarker candidates, that has the opportunity to exchange immunohistochemical identity of patients with protein sequencing assay technology.

**Figure 5:**
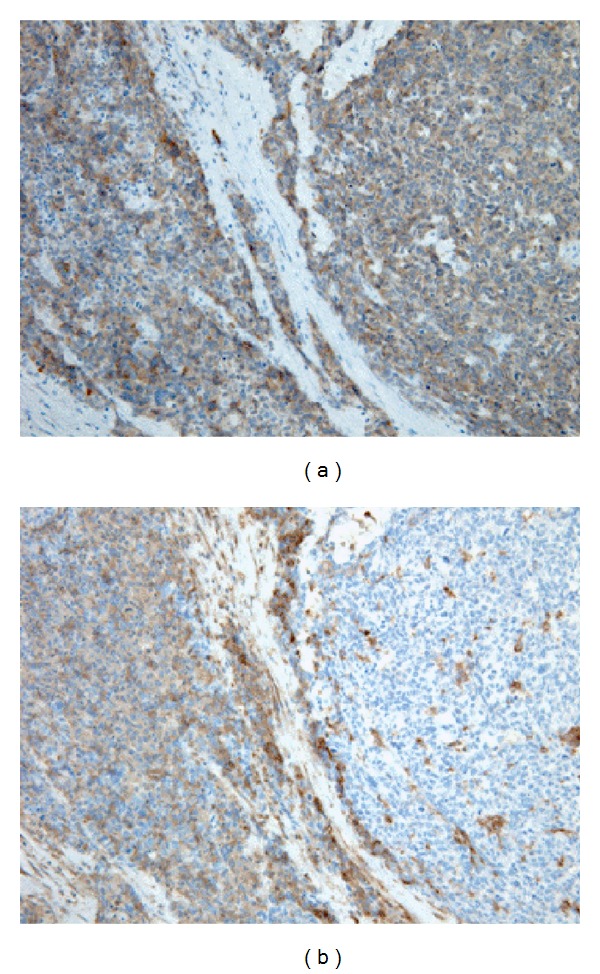
Immunohistochemical staining of LCNEC tissue for (a) Stathmin and (b) for major vault protein (MVP).

Histology still remains the standard for pathology staging, used as a golden standard for protein biomarker diagnosis and proteomics [39], why the validation of the discovered biomarkers was run on additional LCNEC patients. The corresponding immunohistochemical identities were confirmed in these histology studies, shown in Figure 5.

## 5. Drug Localization in Tissue Compartments by Maldi Drug Imaging

The basic understanding of the disease developments and the pathophysiological landmarks that these changes leave is the starting point for an understanding of the molecular fingerprint that these disease stages establish. *In vivo* disease models developed for a given disease state, or rather a mimic of a patient group biology occurrence, are currently a rapidly growing research area. The translation from *in vivo* animal disease models and the predictive values in a human clinical study setting is a critical phase of drug development, establishing the patient dose levels.

**Figure 6:**
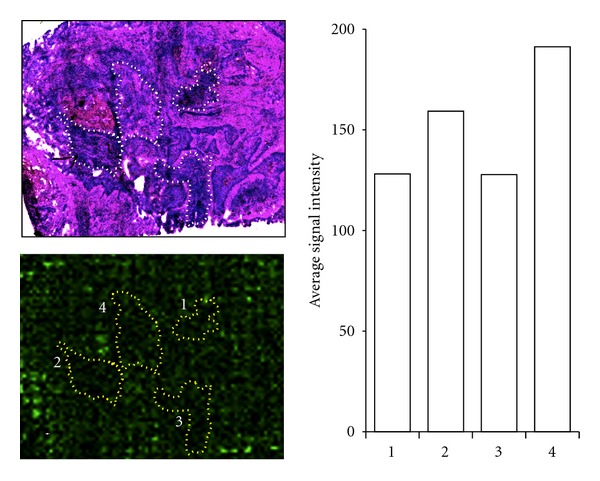
Enlarged region of a squamous cell lung tumor section with MALDI-MS read out of the Erlotinib fragment ion (*m*/*z*  336.19) and HE stained histological details. Typical areas of tumor cells are indicated with yellow-dashed lines.

Drug localization after administration is highly valuable clinical information, where both target specificity and redundancy can be determined. An understanding of the pharmacokinetic as well as pharmacodynamic properties of a drug is of mandatory importance in drug development processes. Imaging technologies are gaining more power in drug characterization, utilizing positron emission tomography (PET), MRI, MS-CT, and MALDI imaging. In order to increase the basic knowledge about drug substances and their pharmacological effects, MALDI-imaging model-based developments have been introduced lately, which includes cell based as well as tissue-based validations of drug compounds [40]. The distribution of TARCEVA in tumor tissues isolated from a lung cancer patient is illustrated in Figure 6. 

## 6. Conclusions

It is envisioned that the upcoming generation of personalized drugs for targeted and stratified patient treatment will break through in major disease areas such as lifestyle-related cancers, in particular lung cancers that have the highest mortality including a predisposing disorder chronic obstructive pulmonary disease. Other cancers such as, for instance; breast, colon, malignant melanoma, and brain are expected to have targeted treatments in the coming years. In addition, cardiovascular diseases, neurodegenerative diseases such as multiple sclerosis (MSc), Parkinson, and Alzheimer, obesity, and diabetes are additional disease areas where a multitude of pipeline drugs are expected to make it as products into the market, and the shelves of the local pharmacy, readily available for patients. Mass spectrometric technologies can provide the “phenotypic fingerprint” required for the concept of personalized medicine. Mass-spectrometry-driven target biomarker diagnoses in combination with high-resolution computed tomography can provide a critical pathway initiative facilitated by a fully integrated e-Health infrastructure system.

##  Expert Commentary

Targeted drugs, as the new generation of medicines, are expected to be used in combination with diagnostics, in order to increase efficient treatment of patients. It is also to be expected that the strong increase in biomarker developments will play a significant role in these strategic changes that will be of huge benefits to the health care as well as to the society and taxpayers. With technology developments associated to the personalized treatment, the clinical chemistry unit of future hospitals will also be making an increased number of analysis and assays in order to provide stronger diagnosis basis for the doctors at hospitals to make improved disease diagnosis of the patient.

We will also see a fast development of higher throughput diagnostic assays, where the breakthrough of the human genome and the human proteome will be used as the basis for these developments.

##  A Five-Year View

We do expect that during a five-year period we have reached a point where protein-based biopharmaceuticals or biologicals have taken a larger percentage of the drug market with a targeted drug approach. Improved clinical chemistry diagnosis with gene- and protein-sequence-based assays will also become state of the art in future medical health care that will result in a much higher number of overall measuring points. High-throughput multiplexed biomarker assay platforms will play an important clinical role as becoming a complement to traditional immunoassays for future use in clinical health care and targeted medicine. The introduction of new biomarkers of tumors, emphysema, and inflammatory reaction in the lung will assist in early identification of disease and in monitoring the effect of therapeutic agents on disease progression.

##  Key Issues

- Within lung cancer, there have been reports on early indication of somatic mutation appearances within the EGF receptor, where personalized drugs in Japan hve been proven highly efficient.
- Personalized medicine is more cost-effective for society, with an overall benefit for the patients as well as for the tax payers.
- Japan has declared a pricing strategy that includes request for new biomarker diagnostics that can be used for patient stratification.
- We will also see a fast development of higher throughput diagnostic assays, where this breakthrough will become a new milestone in modern healthcare.

## Abbreviations

2-DE: Two-dimensional gel electrophoresis
COPD: Chronic obstructive pulmonary disease
CT: Computer tomography
EGFR: Epidermal growth factor receptor
EMEA: European Medicines Agency
FDA: Food and Drug Administration
FFPE: Formalin-fixed paraffin embedding
IHC: Immuno-histochemistry
ILD: Intestinal lung disease
LCa: Lung cancer
LCC: Large cell carcinoma
LC-MS: Liquid chromatography-mass spectrometry
LCNEC: Large cell neuroendocrine lung cancer
MALDI-MSI: Matrix-assisted laser desorption/ionization mass spectrometric imaging
MHLW: Ministry of Health, Labour and Welfare
MRI: Magnetic resonance imaging
MRM: Multiple reaction monitoring
MSc: Multiple sclerosis
NSCLC: Non-small cell lung cancer
PAC: Post-operative adjuvant chemotherapy
PET: Positron emission tomography
SCLC: Small cell lung carcinoma
TKI: Tyrosine kinase inhibitor
WHO: World Health Organization.

## References

[1] Kaneko R (2009). The society created by the longevity revolution–historical development and associated issues. *Japanese Journal of Population*.

[2] Kato H, Nishimura T, Hirano T (2011). A clinician view and experience of proteomic studies in the light of lung cancer in Japanese healthcare. *Journal of Proteome Research*.

[3] Thatcher N, Chang A, Parikh P (2005). Gefitinib plus best supportive care in previously treated patients with refractory advanced non-small-cell lung cancer: results from a randomised, placebo-controlled, multicentre study (iressa survival evaluation in lung cancer). *Lancet*.

[4] Kudoh S, Kato H, Nishiwaki Y (2008). Interstitial lung disease in japanese patients with lung cancer: a cohort and nested case-control study. *American Journal of Respiratory and Critical Care Medicine*.

[5] Davis JC, Furstenthal L, Desai AA (2009). The microeconomics of personalized medicine: today’s challenge and tomorrow’s promise. *Nature Reviews Drug Discovery*.

[6] Hornberger J, Cosler LE, Lyman GH (2005). Economic analysis of targeting chemotherapy using a 21-gene rt-pcr assay in lymph-node-negative, estrogen-receptor-positive, early-stage breast cancer. *American Journal of Managed Care*.

[7] Lutter R, McWilliam A, Nardinelli C (2006). *Health Care Savings from Personalizing Medicine Using Genetic Testing: The Case of Warfarin*.

[8] Keshishian H, Addona T, Burgess M, Kuhn E, Carr SA (2007). Quantitative, multiplexed assays for low abundance proteins in plasma by targeted mass spectrometry and stable isotope dilution. *Molecular and Cellular Proteomics*.

[9] Jaffe JD, Keshishian H, Chang B, Addona TA, Gilette MA, Carr SA (2008). Accurate inclusion mass screening: a bridge from unbiased discovery to targeted assay development for biomarker verification. *Molecular and Cellular Proteomics*.

[10] Addona TA, Abbatiello SE, Schilling B (2009). Multi-site assessment of the precision and reproducibility of multiple reaction monitoring-based measurements of proteins in plasma. *Nature Biotechnology*.

[11] Johnson DH, Fehrenbacher L, Novotny WF (2004). Randomized phase ii trial comparing bevacizumab plus carboplatin and paclitaxel with carboplatin and paclitaxel alone in previously untreated locally advanced or metastatic non-small-cell lung cancer. *Journal of Clinical Oncology*.

[12] Sandler A, Gray R, Perry MC (2006). Paclitaxel-carboplatin alone or with bevacizumab for non-small-cell lung cancer. *New England Journal of Medicine*.

[13] Lynch TJ, Bell DW, Sordella R (2004). Activating mutations in the epidermal growth factor receptor underlying responsiveness of non-small-cell lung cancer to gefitinib. *New England Journal of Medicine*.

[14] Peták I, Schwab R, Örfi L, Kopper L, Kéri G (2010). Integrating molecular diagnostics into anticancer drug discovery. *Nature Reviews Drug Discovery*.

[15] Johnson BE, Jänne PA (2005). Selecting patients for epidermal growth factor receptor inhibitor treatment: a fish story or a tale of mutations?. *Journal of Clinical Oncology*.

[16] Paez JG, Jänne PA, Lee JC (2004). Egfr mutations in lung, cancer: correlation with clinical response to gefitinib therapy. *Science*.

[17] Mok TS, Wu YL, Thongprasert S (2009). Gefitinib or carboplatin-paclitaxel in pulmonary adenocarcinoma. *New England Journal of Medicine*.

[18] Marko-Varga G, Ogiwara A, Nishimura T (2007). Personalized medicine and proteomics: lessons from non-small cell lung cancer. *Journal of Proteome Research*.

[19] Hirano T, Kato H, Maeda M (2005). Identification of postoperative adjuvant chemotherapy responders in non-small cell lung cancer by novel biomarker. *International Journal of Cancer*.

[20] Committee declaration of the drug pricing within the Ministry of Health Labour and Welfare (MHLW).

[21] Raghu G, Nyberg F, Morgan G (2004). The epidemiology of interstitial lung disease and its association with lung cancer. *British Journal of Cancer*.

[22] Azuma A, Kudoh S (2007). High prevalence of drug-induced pneumonia in japan. *Japan Medical Association Journal*.

[23] Koo LC, Clark JA, Quesenberry CP (2005). National differences in reporting ’pneumonia’ and ’pneumonia interstitial’: an analysis of the who international drug monitoring database on 15 drugs in nine countries for seven pulmonary conditions. *Pharmacoepidemiology and Drug Safety*.

[24] Inoue A, Saijo Y, Maemondo M (2003). Severe acute interstitial pneumonia and gefitinib. *Lancet*.

[25] Kudoh S, Takeda K, Nakagawa K (2006). Phase iii study of docetaxel compared with vinorelbine in elderly patients with advanced non-small-cell lung cancer: results of the west japan thoracic oncology group trial (WJTOG 9904). *Journal of Clinical Oncology*.

[26] Ando M, Okamoto I, Yamamoto N (2006). Predictive factors for interstitial lung disease, antitumor response, and survival in non-small-cell lung cancer patients treated with gefitinib. *Journal of Clinical Oncology*.

[27] Danson S, Blackhall F, Hulse P, Ranson M (2005). Interstitial lung disease in lung cancer: separating disease progression from treatment effects. *Drug Safety*.

[28] Nishimura T, Nomura M, Fukuda T, Marko-Varga G, Laurell T MRM MS-based assays to identify biomarkers for lung carcinoma of large-cell neuroendocrine (LCNEC).

[29] Ogiwara A, Kawakami T, Nagasaka K, Marko-Varga G, Laurell T Keys to success for large-scale proteomics analysis conducted with a clinical study.

[30] Nyberg F, Ogiwara A, Harbron CG (2011). Proteomic biomarkers for acute interstitial lung disease in gefitinib-treated japanese lung cancer patients. *Plos one*.

[31] Nomura M, Fukuda T, Fujii K (2011). Preferential expression of potential markers for cancer stem cells in large cell neuroendocrine carcinoma of the lung. An FFPE proteomic study. *Journal of Clinical Bioinformatics*.

[32] Brambilla E (1999). Who 1999 classification of lung cancers: a guided tour. *Annales De Pathologie*.

[33] Prieto DA, Hood BL, Darfler MM (2005). Liquid tissue: proteomic profiling of formalin-fixed tissues. *Biotechniques.*.

[34] Hood BL, Conrads TP, Veenstra TD (2006). Unravelling the proteome of formalin-fixed paraffin-embedded tissue. *Briefings in Functional Genomics and Proteomics*.

[35] Hood BL, Conrads TP, Veenstra TD (2006). Mass spectrometric analysis of formalin-fixed paraffin-embedded tissue: unlocking the proteome within. *Proteomics*.

[36] Picotti P, Rinner O, Stallmach R (2010). High-throughput generation of selected reaction-monitoring assays for proteins and proteomes. *Nature Methods*.

[37] Surinova S, Schiess R, Hüttenhain R, Cerciello F, Wollscheid B, Aebersold R (2011). On the development of plasma protein biomarkers. *Journal of Proteome Research*.

[38] Anderson NL, Anderson NG, Pearson TW (2009). A human proteome detection and quantitation project. *Molecular and Cellular Proteomics*.

[39] Végvári Á, Marko-Varga G (2010). Clinical protein science and bioanalytical mass spectrometry with an emphasis on lung cancer. *Chemical Reviews*.

[40] Marko-Varga G, Fehniger TE, Rezeli M, Döme B, Laurell T, Végvári Á (2011). Drug localization in different lung cancer phenotypes by maldi mass spectrometry imaging. *Journal of Proteomics*.

